# Complications Related to Childhood Idiopathic Nephrotic Syndrome, Its Treatment and the Associated Risks in Patients

**DOI:** 10.7759/cureus.43929

**Published:** 2023-08-22

**Authors:** Deniz Karakaya, Tülin Güngör, Evrim Kargın Çakıcı, Fatma Yazilitaş, Evra Çelikkaya, Mehmet Bülbül

**Affiliations:** 1 Pediatric Nephrology, Dr Sami Ulus Maternity and Child Health and Diseases Training and Research Hospital, Ankara, TUR; 2 Pediatric Nephrology, Ankara Etlik City Hospital, Ankara, TUR

**Keywords:** chronic renal diseases, long standing complication, complication of treatment, idiopathic nephrotic syndrome, childhood onset

## Abstract

Aim

Nephrotic syndrome is the most common childhood glomerular disorder, but data on the associated complications are limited and predisposing risk factors have not been fully defined. The aim of this study was to evaluate disease- and treatment-related acute and chronic complications in patients with childhood idiopathic nephrotic syndrome (INS), and to identify the risk factors involved in the development of complications.

Methods

This single-center study was performed at the pediatric nephrology department of a tertiary pediatric hospital in Turkey. The study included 411 patients with a diagnosis of childhood INS, 128 of whom had disease-related and treatment-related complications. Patients diagnosed and followed-up between January 2010 and January 2022 were evaluated retrospectively.

Results

Complications occurred in 31.1% of the 411 patients. Mean age at the time of diagnosis was 7.54 ± 4.37 years, and the male/female ratio was 0.9:1. Among the patients with complications, 96.9% were disease-related, and 50.8% were treatment-related complications. In older age, high proteinuria level, a low estimated glomerular filtration rate (eGFR) level at diagnosis, and female gender were significant risk factors for complication development (P = 0.000, P = 0.006, P = 0.04, and P = 0.07, respectively). Chronic kidney disease (CKD) developed in 7% of patients and 2.9% of patients had end-stage renal disease (ESRD). Additionally, three of 12 patients with progressive ESRD underwent transplantation. Also the incidence of ESRD was significantly higher in the patients with complications than in those without complications (P < 0.05).

Conclusion

The present findings suggest that careful monitoring of patients with childhood INS at risk for complications and implementation of personalized treatment programs can improve long-term outcomes, especially in patients that progress to ESRD and are followed-up with dialysis or transplantation as targeted therapy.

## Introduction

Nephrotic syndrome (NS) is the most common glomerular disorder of childhood, with an incidence of 2-7 per 100,000 children [1]. Clinically, it is defined as the presence of the four classic symptoms: massive proteinuria, hypoalbuminemia, diffuse edema, and, in most cases, hyperlipidemia. The main causes of childhood ‘idiopathic’ NS are minimal change disease (MCD) and, less frequently, focal segmental glomerulosclerosis (FSGS).

The pathogenesis of NS involves structural changes in the glomerular filtration barrier, increased permeability, and consequent massive leakage of albumin and other negatively charged proteins into urine [2]. Loss of plasma proteins in urine causes complications of NS, either as a direct result of varying protein concentrations in plasma or as a secondary consequence of altered cellular function [3]. Complications of childhood NS fall into two categories: disease-related complications treatment-related complications.

Data on complications in pediatric NS patients are limited and predisposing risk factors are yet to be fully defined. The aim of the present study was to use our large patient data set to evaluate treatment- and disease-related acute and chronic complications in children with idiopathic nephrotic syndrome (INS), and to identify the risk factors involved in the development of complications. It was hypothesized that careful monitoring of patients at risk for complications and implementation of personalized treatment programs would improve long-term outcomes.

## Materials and methods

Study population

This single-center retrospective study was performed at the pediatric nephrology department of a tertiary pediatric hospital in Turkey. The study included 411 patients with a diagnosis of childhood INS, 128 of whom had disease-related and treatment-related complications. Patients diagnosed and followed-up between January 2010 and January 2022 were evaluated retrospectively. Inclusion criteria were age 1-18 years and regular follow-up at the pediatric nephrology department for ≥1 years. Patients diagnosed with infantile and secondary NS (immunoglobulin A (IgA) nephropathy, lupus, Henoch Schönlein Purpura, or malignancy) were excluded from the study.

Physical examination and laboratory findings for all patients with NS were reviewed. Demographic data, clinical pattern of NS, renal biopsy results, hospitalizations, presence of infection, history of venous thromboembolism (VTE), and treatment-related complications were recorded. Duration of follow-up, physical examination findings, including edema, blood pressure (BP), and laboratory findings, such as serum creatinine, estimated glomerular filtration rate (eGFR), albumin, and urinalysis, were retrospectively obtained from patient medical records and reviewed. Kidney biopsy was performed in patients aged <1-10 years at disease onset that had accompanying macroscopic hematuria and hypertension (HT), were resistant to steroids, or had a decreased glomerular filtration rate.

Definitions

Kidney Disease Improving Global Outcomes (KDIGO) 2012 guidelines were used as the reference for clinical diagnosis of NS, definitions of remission/relapse, and acute kidney injury (AKI). The treatment protocol was also based on KDIGO recommendations [4]. NS was defined as edema with massive proteinuria (greater than 40 mg/m^2^ per hour and/or loss of 3 g or more per day of protein into the urine or, on a single spot urine collection, the presence of 2 g of protein per gram of urine creatinine) and serum albumin <2.5 g/dl.

eGFR was calculated according to the Schwartz formula [5]. Chronic kidney disease (CKD) was defined as a creatinine clearance <60 ml/min/1.73 m^2^ in two consecutive measurements performed ≥3 months apart [4]. AKI was defined according to the KDIGO guidelines as stage 2 or 3 AKI, i.e., a two-fold increase in the serum creatinine level from baseline. We used the baseline creatinine value, but if it was unknown the value at remission was accepted as the standard. Thrombosis was diagnosed via Doppler ultrasonography or CT/MR angiography. Complications related to massive proteinuria/hypoalbuminemia are given in Table 1.

**Table 1:** Complications related to massive proteinuria or hypoalbuminemia URTI: Upper respiratory tract infection; UTI: Urinary tract infection; AKI: Acute kidney injury

Infections	
Bacterial	Peritonitis
	Cellulitis/abscess
	Sepsis/bacteremia
	Pneumonia
	Meningitis
	UTIs
	Gastroenteritis
	URTI
Viral	Chicken pox
	Hepatitis
	URTI
Hypovolemia and AKI	
Thromboembolic complications	
Venous	Deep veins in the extremities
	Cerebral venous sinuses
	Renal vein
	Jugular subclavian vein
	İntracardiac
Arterial	Pulmonary
	Cerebral
	Extremities
Loss of binding proteins	Hypocalcemia (vitamin D-binding globulin)
	Hypothyroidism (thyroid-binding protein)
	Anemia (transferrin)
	Hyperlipidemia
	Cardiovascular disease

HT was diagnosed according to the American Academy of Pediatrics 2017 HT Guidelines and defined as the mean of three consecutive systolic and/or diastolic BP measurements via the auscultatory method ≥95th percentile for age, gender, and height, or BP >130/80 mm Hg in participants aged ≥13 years [6]. A Z score <-2.0 for age and gender in the bone mineral density (BMD) test was defined as a decrease in BMD, and osteoporosis was diagnosed in patients with a history of fracture. Psychosis secondary to steroid therapy was defined as any change in mental status detected by the clinician. Cushingoid appearance and obesity, defined as complications, include patients who developed completely after treatment and were therefore considered drug-related. At the beginning of the treatment, the patients were informed about the complications, nutritional recommendations were made and they were kept under regular control. The classification of treatment-related complications is shown in Table 2. The study protocol was approved by the Ethics Committee and was performed in accordance with the Declaration of Helsinki (2020-KAEK-141/370).

**Table 2:** Complications due to the side-effects or toxicity of drugs used to treat NS NS: Nephrotic syndrome; BMD: Bone mineral density; ICP: Increased intracranial pressure; HT: Hypertension

Corticosteroids	Cushingoid features
	Growth retardation
	HT
	Osteoporosis
	Decreased BMD
	Posterior subcapsular cataracts
	Pseudotumor cerebri (ICP)
	Glaucoma
	Behavior disorders
	Peptic ulcer
	Skin disorders
	Glucose intolerance
Cyclophosphamide	Alopecia
	Hemorrhagic cystitis
	Bone marrow suppression
	Gonadal toxicity
	Malignancy
	Nausea
	Infection
Mycophenolate	Bone marrow suppression
	Gastrointestinal complaints
	Infection
Cyclosporine	Hirsutism
	Gingival hypertrophy
	HT
	Nephrotoxicity
	Neurotoxicity
Tacrolimus	Diabetes
	Nephrotoxicity
	HT
	Tremor
	Headache
Rituximab	Bronchospasm
	Leukoencephalopathy
	Viral reactivation

Statistical analysis

Data were analyzed using IBM SPSS Statistics for Windows v.22.0 (IBM Corp., Armonk, USA). The Kolmogorov-Smirnov test was used to determine the normality of the distribution of data. Parametric variables are shown as mean ± SD, and nonparametric variables are shown as median (inter quartile range (IQR)). Categorical variables are presented as number and percentage. Student’s t test for independent samples was used to compare the means of continuous variables. The χ2 test or Fisher’s exact test was used to compare categorical variables. Logistic regression analysis was used to identify variables that were independently associated with complications at the 95% CI. Variables selected for multivariate analysis were used to build a final model after discarding any violation of proportionality assumptions. The level of statistical significance was set at P < 0.05.

## Results

Complications occurred in 128 (31.1%) of the patients, of which approximately 36% had frequent relapsing/steroid-dependent NS. Mean age at diagnosis of NS was 7.54 ± 4.37 years, and the male to female ratio was 0.9:1. In total, 61.7% of the patients were diagnosed histopathologically and 30.5% had FSGS. Among the patients with complications, 96.9% were disease-related, and 50.8% were treatment-related complications. Only 3.1% of the patients had treatment-related complications without disease-related complications. The most common complication was infection, with a rate of 63.3%. At the time of presentation, the mean eGFR was 110.23 ± 43.27 mL/min/1.73 m-2, the mean albumin level was 1.88 ± 0.85 mg/dL, and the mean proteinuria level was 222.81 ± 134.16 mL/min/1.73 m-2. Mean duration of follow-up was 6.65 ± 4.18 years.

 Among the infectious complications, 37% were upper respiratory tract infections (URTIs), followed by pneumonia (23.4%), chickenpox (21%), peritonitis (18.5%), gastroenteritis (18.5%), urinary tract infection (UTI) (11.1%), cellulitis (6.2%), bacteremia (3.7%), and hepatitis B infection (3.7%). In all, 49.4% of the patients had a history of >1 infections, and 33.3% presented with infection at the time of NS diagnosis. Additionally, 86.4% of the patients had ≥1 hospitalizations due to infection. At the time of infectious complications, 32 (39.5%) patients were not receiving immunosuppressive therapy and 55 (67.9%) were in relapse.

Acute renal failure developed in 12.1% of patients, and 90% of these events occurred during hospitalization. AKI occurred a mean 2.90 ± 2.96 years after NS diagnosis. During AKI, 20 (40%) of the patients were not receiving immunosuppressive therapy, and 29 (58%) patients were diagnosed with AKI during relapse.

Thromboembolic complications developed in 3.8% of the patients, of which nine (56.3%) of the cases were detected during hospitalization and one (6.3%) of the patients required intensive care. At the time of thrombosis, six (37.5%) patients were not receiving immunosuppressive therapy and 12 (75%) patients were in relapse. Among the 16 patients with thrombosis, seven (43.7%) had deep vein thrombosis (DVT) of the femoral, popliteal, and cephalic veins of the lower extremities; five (31.25%) had catheter-related thrombosis in the jugular or subclavian vein; two (12.5%) had renal vein thrombosis; one (6.25%) had sagittal sinus thrombosis and associated cerebral infarct; and one (6.3%) had intracardiac (in both the right ventricle and right atrium) thrombosis. Diagnosis of NS and thrombosis occurred at the same time in one (6.3%) patient. Thrombosis occurred during the first year following diagnosis of NS in nine (56.25%) of the patients and >1 year after diagnosis in seven (43.75%). Thrombosis was observed in more than one region in 18.75% of patients with thrombosis. In patients with loss of binding proteins, 9.2% had anemia, 2.6% had hypothyroidism, and 3.1% had hypocalcemia as complications. Hyperlipidemia as a cardiovascular complication occurred in 21.1% of the patients.

Treatment-related complications occurred in 15.5% of the INS patients. Complications related to ≥1 drugs were observed in 26 patients. Corticosteroid-related complications occurred most frequently (89.2%). Corticosteroid-related cushingoid features (obesity) were observed after treatment in 12 patients, growth retardation in 15, HT in three, low BMD in 30 (osteoporosis: n = 2), posterior subcapsular cataracts in 27, glaucoma in three, behavioral disorders in one, increased intracranial pressure (ICP) in one, gastrointestinal side-effects (peptic ulcer) in 11, skin disorders in 15, and glucose intolerance in three. In addition, 32 (55.2%) patients had corticosteroid-related complications affecting ≥2 systems. Alkylating agent-related complications were noted in 6.2% of the patients, of which infectious complications developed in 3 and nausea in one. Cyclosporine A-related complications occurred in 22 (33.8%) patients, as follows: nephrotoxicity: n = 11; hirsutism: n = 10; HT: n = 4; neurotoxicity: n = 2. Four of the patients who developed cyclosporine-related complications had more than one cyclosporine-related complication. Mycophenolate mofetil-related complications developed in three (4.6%) patients, of which two had gastrointestinal system complaints, one had marrow suppression, and one had infection. Tacrolimus-related complications occurred in eight (12.3%) patients as follows: nephrotoxicity: n = 4; diabetes mellitus: n = 1; HT: n = 1; tremor: n = 1; headache: n = 1. Only one of the five patients treated with rituximab had bronchospasm during infusion. The distribution of complications is shown in Table 3.

**Table 3:** Distribution of patients with complications AKI: Acute kidney injury

Complication Releated with Disease	N=124
Infections	81
Hypovolemia and AKI	50
Thromboembolic complications	16
Hypocalcemia	13
Hypothyroidism	11
Anemia	38
Hyperlipidemia	87
Complication related with treatment	N=64
Corticosteroids	57
Cyclosporine	22
Tacrolimus	8
Cyclophosphamide	4
Mycophenolate	3
Rituximab	1

There wasn’t a significant difference in the albumin level between the patients with and without complications (P = 0.33). The level of proteinuria was significantly higher in the patients with complications (P = 0.02). Patients with complications had a significantly lower eGFR at admission (P = 0.04), were significantly older at the time of diagnosis (P = 0.00), and were female significantly more frequently (P = 0.002) (Table 4). Logistic regression analysis showed that in older age at diagnosis, female gender, a high proteinuria level, and a low eGFR were significant risk factors for complication development (P = 0.000, P = 0.006, P = 0.04, and P = 0.07, respectively). The logistic regression analysis results are shown in Table 5.

**Table 4:** Characteristics of the patients with and without complications eGFR: Estimated glomerular filtration rate

	With Complications N=128	Without Complications N=283	P
Female gender, n (%)	61 (47.7)	89 (31.4)	0.00
Age, years, mean ± SD	7.54 ± 4.3	5.25 ± 3.2	0.00
eGFR, mL·min^–1^·1.73 m^–2^, mean ± SD	110.23 ± 43.2	118.24 ± 18.2	0.04
Proteinuria, mg·m^–2^·h^–1^, mean ± SD	222.81 ± 134.1	186.75 ± 140.6	0.02
Serum albumin, gdL^–1^, mean ± SD	1.88 ± 0.8	1.81 ± 0.5	0.33

**Table 5:** Logistic regression analysis of the risk factors for the development of complications of NS CI: Confidence interval; OR: Odds ratio; NS: Nephrotic syndrome; eGFR: Estimated glomerular filtration rate

	Univariate Analysis			Multivariate Analysis		
Parameter	Odds Ratio	95% CI	P	OR	95% CI	P
Gender (F)	1.985	1.293-3.045	0.002	1.872	1.197-2.928	0.006
Presentation age, years	1.168	1.104-1.236	0.000	1.163	1.099-1.232	0.000
Presentation albumin, gdL^–1^	1.161	0.859-1.569	0.333	1.096	0.769-1.562	0.612
Presentation proteinuria, mg·m^–2^·h^–1^	1.002	1.000-1.004	0.032	1.001	0.999-1.003	0.040
Presentation eGFR, mL/min/1·1.73 m^2^	0.990	0.982-0.997	0.010	0.993	0.984-1.002	0.078

During the long-term follow-up, CKD developed in 7% of the patients and the mean time from diagnosis to CKD was 4.19 ± 4.78 years. In total, 2.9% of the patients had end-stage renal disease (ESRD). Moreover, three of the 12 patients with progressive ESRD underwent transplantation. Furthermore, 83.3% of the patients that progressed to ESRD had disease- or treatment-related complications. As compared to the patients without complications, those with complications had a significantly higher incidence of ESRD (P < 0.05).

## Discussion

NS is among the most common glomerular diseases of childhood, and is characterized by severe and prolonged proteinuria, and prolonged and multiple treatments that are associated with an increased risk of systemic complications. The present study aimed to evaluate disease- and treatment-related complications, and the associated risk factors in patients with childhood INS. The remarkable result of our study is that the risk factors identified for complications associated with INS are presented. The risk factors for the development of complications in these patients were determined as in older age at diagnosis, female gender, a high proteinuria level, and a low eGFR were significant risk factors.

 Patients with NS are at risk of infection. Despite treatment advances, infection remain a major problem in developing countries and a cause of death [7]. NS is associated with a low IgG concentration due to urinary loss and altered production, and the presence of factor B and factor I in alternative pathway components, which contribute to the risk of infection [7]. In the present study the most common complication observed during follow-up was URTIs (37%). Similarly, other studies report that acute respiratory tract infection is the most common infection [8]. In terms of the frequency of complications in the present study URTIs were followed by pneumonia, chickenpox, peritonitis, gastroenteritis, UTI, cellulitis, bacteremia, and hepatitis B infection. Pneumonia frequency had the variability in the studies and observed in 3.3%-12.9% of cases [9,10]. Peritonitis is also a life-threatening and common infectious complication of NS and it was reported that peritonitis had a high rate among the infectious complications, as in the present study [9,10]. Adedoyin et al. showed that the one of most common infectious complications of NS is UTI with incidence of 16% similarly with this study [11]. Studies have reported a low incidence of skin infections in children with NS and in the present study the frequency of cellulitis and skin infections was only 6.2% [12]. In the present study the bacteremia, hepatitis, and chickenpox rates were similar to that of skin infections, although they are rarely reported in the literature [13]. Based on the present findings, we think that the diversity of infectious complications may related to demographic (geographic and socioeconomic) characteristics.

Little is known about AKI in children with NS. Among patients with AKI, most of them have a history of intravascular volume depletion, including diarrhea, vomiting, and dehydration. In addition, infections and drug use are also known as major predisposing factors underlying AKI [14]. In the present study 12.1% of the patients developed AKI and 90% of these cases occurred during hospitalization. Sato et al. reported that 24% of INS patients in their study had AKI [15]. Similarly, Kim et al. showed a high incidence of AKI (32.2%) among children hospitalized with NS in their single-center studies [16].

Thrombosis is a well-known complication of NS that is associated with a high risk of mortality. Various mechanisms have been reported to promote thrombosis in NS patients that generally fall into two categories: urinary loss of proteins that prevent thrombosis and increased synthesis of factors that promote thrombosis. In the present study, thromboembolic complications were observed in 3.8% of the patients. In addition, 12 (75%) patients were in relapse at the time they had thrombosis. Among the 16 patients with thrombosis, the most common type was catheter-related thrombosis in the jugular and subclavian veins (n = 5 (31.25%)), and DVT of the femoral, popliteal, and cephalic veins of the extremities (n = 7 (43.7 %)). The incidence of thrombosis in the present study is similar to the overall rate of 1.8%-6.6% reported earlier [17]. As reported earlier, in the present study thrombosis occurred during the relapse phase of NS in most patients [17,18]. Lilova et al. showed that the most common sites of thrombosis are the DVT and central venous thrombosis as in the present study [18].

It is known that such complications as anemia, hypothyroidism, and hypocalcemia can occur in NS patients due to loss of binding proteins. In the present study, anemia was observed in 9.2% of patients, hypothyroidism in 2.6%, and hypocalcemia in 3.1%. According to the literature, the prevalence of iron deficiency anemia in nephrotic patients is variable (19.2%-59%) [19,20]. Thyroid functions were evaluated in children with NS and hypothyroidism was noted in 58.6% of patients [21]. We think the difference in the hypothyroidism and anemia rate may be due to differences in the criteria used to definations and inclusion. A study on the relationship between NS and hypocalcemia based on measurement of ionized calcium reported that 3% of patients had hypocalcemia [22]. Studies on the frequency of hypocalcemia in NS patients are limited, but they all report a very low rate when based on ionized calcium.

Treatment-related complications vary greatly by type and frequency in NS patients due to a variety of treatments, drug side-effects, and metabolic response. In the present study corticosteroid-related complications were the most common (89.2%). Low BMD (n = 30) and posterior subcapsular cataracts (n = 27) were the most common corticosteroid-related complications. Oh et al. observed NS patients treated with steroids, and they found HT being the most common complication, affecting 62% of patients [23]. A small-scale Japanese study reported that 17 (40.5%) of 42 patients treated with steroids for primary kidney disease developed corticosteroid-induced diabetes [24]. In studies reported from centers where routine ophthalmologic evaluation is performed, steroid-associated cataracts has been shown to occurred in 10%-27% of children with NS [25]. Most likely, the variation in outcomes can be explained by differences in the duration of steroid therapy and cumulative steroid dose, or by clinicians' management strategies for complications.

Large-scale studies presenting complications associated with non-steroidal immunosuppressive agents are very limited. Complications associated with alkylating agents developed in 6.2% of the present study’s patients, as infectious complications in three patients and nausea in one patient. Complications with cyclosporine A developed in 22 patients, with 11 with nephrotoxicity, 10 with hirsutism, four with HT, and two with neurotoxicity. Complications with tacrolimus developed in four patients, including four cases of nephrotoxicity, one of diabetes mellitus, one of HT, one of tremor, and one of headache. Mycophenolate mofetil-related complications developed in three patients, as follows: gastrointestinal system complaints: n = 2; bone marrow suppression; n = 1; infection: n = 1. Only one of the five patients treated with rituximab had bronchospasm during infusion. A study that included 47 NS patients treated with alkylating agents observed the following adverse effects: leukopenia (n = 1); acute chemical cystitis (n = 1); alopecia (n = 1); severe infections (n = 1) [26]. Another study reported that 6% of NS patients treated with cyclosporine A had renal failure and 10% had HT, which is similar to the present findings. A study on the adverse effects of tacrolimus in childhood NS patients observed that the most common adverse event was diarrhea, followed by AKI, hyperglycemia, and HT [27]. Also a study on NS patients treated with mycophenolate mofetil reported abdominal pain as an adverse reaction in two patients [28]. Rituximab is generally well tolerated in most childhood NS patients and the most commonly reported adverse reactions are infusion-related, with a frequency of 5%-53% [29]. The treatment-related side effects and their frequencies noted in the present study are similar to those in the literature.

During the long-term follow-up (mean: 6.65 ± 4.18 years) of the present study’s patients with complications, CKD developed in 22.7% and ESRD developed in 9.4%. As compared to the patients without complications, those with complications had a significantly higher incidence of ESRD. These rates are also higher than in earlier studies in INS, which reported a CKD prevalence of 3%-10% and an ESRD prevalence of 3.6% during a mean follow-up of 7.70 ± 3.81 years [30]. It supports the conclusion that the development of CKD and ESRD is more common in patients with complications.

The study has some limitations, including its single-center retrospective design. Despite these limitations, we think the study makes a significant contribution to the literature, as it presents for the first time data on the complications of childhood NS and data on both disease- and treatment-related complications, as well as the risk factors for the development of complications. Another limitation of our study is the deficiencies in genetic data. It could not be presented in our study because genetic studies in our patients were insufficient to reach a conclusion. However, it is known that the frequency of ESRD is much higher in patients who are investigated for reasons such as steroid resistance, the patient's age at presentation (infantile-congenital NS), the presence of a family history, and who are shown to have a genetic mutation [31]. For this reason, we think that if genetic results were included in our study, we could obtain much more enlightening results. Therefore, there is a need for larger studies that include clinical and genetic findings.

## Conclusions

In this study, proteinuria levels were found to be significantly higher and eGFR significantly lower in patients who developed complications compared to those who did not. At the same time, patients who developed complications were older at the time of diagnosis and were more often female. With this, advanced age at the time of diagnosis, female gender, high proteinuria level, and low eGFR were identified as significant risk factors for the development of complications.

As a result, early diagnosis and appropriate treatment of acute complications are critically important to the treatment of children with NS. It is crucial to determine the risks of these complications and to increase physician awareness of them, especially for associated with the development of ESRD.
